# Deciphering Prognostic Value of *TTN* and Its Correlation With Immune Infiltration in Lung Adenocarcinoma

**DOI:** 10.3389/fonc.2022.877878

**Published:** 2022-07-08

**Authors:** Jianing Chen, Yaokai Wen, Hang Su, Xin Yu, Ruisheng Hong, Chang Chen, Chunxia Su

**Affiliations:** ^1^School of Medicine, Tongji University, Shanghai, China; ^2^Department of Medical Oncology, Shanghai Pulmonary Hospital & Thoracic Cancer Institute, School of Medicine, Tongji University, Shanghai, China; ^3^Department of Thoracic Surgery, Shanghai Pulmonary Hospital, School of Medicine, Tongji University, Shanghai, China; ^4^Department of Radiation Oncology, First Affiliated Hospital of Soochow University, Suzhou, China

**Keywords:** lung Adenocarcinoma, *TTN*, prognosis, biomarker, NSCLC

## Abstract

**Background:**

Lung adenocarcinoma (LUAD) is the most common type of lung cancer, accounting for around 40%. Despite achievements in the treatment approach, the prognosis is still dismal, with overall survival of fewer than five years. Thus, novel prognostic biomarkers are needed to predict the clinical outcomes of individual patients better. *TTN* has a high mutation rate in the LUAD, which encodes a large abundant protein of striated muscle. However, the value of *TTN* in prognosis and the immune environment are poorly understood.

**Methods:**

We investigated the clinicopathological characteristics, transcriptional and protein level, prognostic value, biological function, and its relationship with immune infiltration of *TTN* gene in LUAD patients through bioinformatics analysis.

**Results:**

*TTN* expression was significantly lower in LUAD than that in normal lung tissue. Lower *TTN* expression was associated with worse survival. Besides, *TTN* is highly expressed in alveolar type 2 cells which were surmised as the origin of LUAD.

**Conclusion:**

Our findings indicated the potential prognostic value of *TTN* and its role as a biomarker for determining the immune infiltration levels in patients with LUAD.

## Introduction

Lung adenocarcinoma (LUAD) accounts for a majority of cancer-related death worldwide. In the last decade, breakthroughs in immunotherapy research have dramatically improved survival rates for several tumor types (1–4), and revolutionized the management of cancer (5). Immunological checkpoint blockers (ICBs) targeting PD-1, PD-L1, and CTLA-4 have been widely used, showing extensive clinical benefits, rapidly expanding to more than a dozen clinical indications (6, 7).

Several clinical trials showed that the amounts and characteristics of immune cells in the immune microenvironment are key predictors of ICBs response. KEYNOTE-086 revealed a positive correlation between the number of TILs and the response to pembrolizumab (8). In the KEYNOTE-137 study, the level of PD-L1 expression and stromal tumor-infiltrating lymphocyte levels (TIL) had a strong correlation with response rates of pathologic complete response in triple-negative breast cancer (9). An inflamed tumor microenvironment, relatively abundant CD8+ cytotoxic T cells, relatively deficient CD4+ regulatory T cells, and activation of interferon-mediated signaling are required for ICBs therapeutic response. Therefore, deciphering the characteristics of tumor microenvironment and searching for immunotherapy biomarkers can help us predict the efficacy of immunotherapy and improve the outcome of treatment.

Titin is a key component in the assembly and functioning of vertebrate striated muscles. It acts a pivotal role in many diseases, like cardiomyopathy, limb-girdle muscular dystrophy, and multiple types of cancer (10, 11). Previous researches have focused on the function of *TTN*-AS1 in diverse cancers (12–16). Titin-antisense RNA1 (*TTN*-AS1) has been regarded as a tumor-promoting lncRNA in numerous cancers, such as LUAD, hepatocellular carcinoma, cervical cancer, papillary thyroid cancer, and gastric cancer (12, 17–21). Besides, *TTN* missense mutation plays a favorable prognostic role in lung squamous cell carcinoma(LUSC) (22). *TTN* is a protein-coding gene with a high mutation rate in LUAD. However, to our knowledge, the role of *TTN* mutation in LUAD is rarely reported.

Although immunotherapies have become the standard of care in patients with advanced LUAD, a fraction of patients still have a poor prognosis. Herein, we conducted comprehensive research to elucidate the prognosis value and function of *TTN* in LUAD, aiming to provide a new biomarker for predicting prognosis and a new immunotherapy target for advanced LUAD patients.

## Methods

### Expression Level and Biological Functions of *TTN* Different Types of Cancers

The expression level of the *TTN* gene in different types of cancers was analyzed in the Oncomine database (23) (https://www.oncomine.org/resource/login.html) and TIMER (24) (http://timer.cistrome.org/). The threshold was determined according to the following values: *P*-value of 0.0001, fold change of 2, gene ranking of top 10% and data type of all.

### Prognosis Analysis

The overall survival and progress-free survival curves correlated to *TTN* expression in LUAD were plotted by Kaplan-Meier plotter (25, 26) (http://kmplot.com/analysis/). The correlation between *TTN* expression and survival in various cancer types was investigated in the PrognoScan database (27) (http://www.abren.net/PrognoScan/). The threshold was adjusted by a log-rank p-value of <0.05. Hazard ratio (HR) with 95% confidence intervals (CI) was also calculated.

### Clinicopathological Characteristics Analysis

Clinicopathological characteristics and the mutation information of *TTN* of LUAD patients were explored in the cBioPortal database (28) (https://www.cbioportal.org). CBioPortal database contains somatic mutation and copy number variation data from the cancer genome atlas (TCGA) database (29) (https://www.cancer.gov/aboutnci/organization/ccg/research/structuralgenomics/tcga). The data of alteration frequency (mutation, fusion, amplification, deep deletion) of *TTN* was also visualized and downloaded from the official website. Besides, we excluded samples that are not profiled for all queried genes in all queried profiles.

### Human Protein Atlas and CancerSEA Database

*TTN* mRNA expression and other related cell type markers in each cell type cluster of lung tissue were visualized by Uniform Manifold Approximation and Projection (UMAP) in the Human protein atlas (30, 31) (HPA; www.proteinatlas.org). Specificity and distribution classification were performed to determine the number of genes elevated in different cell types. The genes expressed in each of the cell types were explored in interactive UMAP plots and bar charts. Color-coding is based on groups of cells, and each cell type has common functional features. The functional state of *TTN* in various cancer types was analyzed by CancerSEA (32) (http://biocc.hrbmu.edu.cn/CancerSEA/). Correlations between the gene of interest and functional state in different single-cell datasets were filtered by correlation strength > 0.3 and *P*-value < 0.05.

### Tumor Immune Infiltration Analysis

The relevance of *TTN* expression to tumor immune infiltration was analyzed *via* TIMER (http://timer.cistrome.org/). The gene expression level was displayed with log2 RSEM. The significantly correlated genes in TIMER were validated in GEPIA (33) (http://gepia.cancer-pku.cn/index.html). The Spearman method was used to analyze the correlation coefficient. *TTN* was used for the x-axis, and other genes of interest are represented on the y-axis. The tumor and normal tissue datasets were used for analysis.

### GEO Database

One independent cohort (GSE116959) was downloaded from the GEO database. GSE116959 contains transcriptome profiling information of 57 LUAD samples and 11 peritumoral normal lung tissues, with gene expression measured by Agilent-039494 SurePrint G3 Human GE v2 8x60K Microarray 039381 in GPL17077. The *TTN* expression of each sample was analyzed using an unpaired t-test.

### Quantitative Real-Time PCR

Two tumor tissue samples and paired tumor-adjacent tissues, five human lung tumor cell lines and one human pulmonary alveolar epithelial cell line (HPAEpiC) were used to detect transcriptional expression of *TTN*. A549, HCC827, H3255 and H1975 cell samples belong to lung adenocarcinoma cell lines, H1703 belongs to lung squamous cell line and HPAEpiC belongs to human pulmonary alveolar epithelial cell line. The mRNA expression of *TTN* was detected by real-time PCR, and GAPDH was the internal control gene. Real‐time PCR was performed with TB Green qPCR Master Mix (RR920A, Takara, Beijing, China) using the QuantStudio 6 Flex Real-Time PCR System (Thermo Fisher Scientific, Waltham, MA, USA). The expression values were analyzed by using 2−ΔΔCt relative quantitative methods. Primer sequences for the real-time PCR were listed as follows:

*TTN* forward: 5′- ACCCTTCTTTGACATCCGT -3′, reverse: 5′- TACTTTCCGCCACTTCGT -3′;

GAPDH forward: 5′- ACAACTTTGGTATCGTGGAAGG -3′, reverse: 5′- GCCATCACGCCACAGTTTC -3′.

### Immunohistochemistry

Clinical samples were obtained from 1 patient with LUAD who was surgically treated at shanghai pulmonary hospital. The LUAD tissue and the paired adjacent tissues were prepared into 5 μm paraffin sections and incubated with mouse polyclonal antibodies of titin (1:150, Sigma, USA) at 4° overnight in a refrigerator. The sections were coupled with the secondary antibody labeled with horseradish peroxidase (1:400, Abcam, USA) at room temperature for 1.5 h, then each section was stained with DAB reagent, and counterstained with hematoxylin. IHC sections were independently reviewed by two pathologists (JC, HS).

### Western Blotting

Protein from five lung cancer cell samples and one normal epithelial cell sample were extracted for used in the following western blotting (WB) experiments. WB experiments were performed according to the detailed protocol previously reported (34), with antibody against titin N-terminal (mouse;1:1000; Sigma SAB1400284).

### Statistical Analysis

Survival curves were generated by the PrognoScan and Kaplan Meier plots. The results generated in PrognoScan were performed with the hazard ratio (HR), 95% confidence interval (CI), and Cox *P*-values. The results generated in Oncomine are displayed with *P*-values, fold changes, and ranks. The results of Kaplan Meier plots and GEPIA are displayed with HR and P or Cox *P*-values from a log-rank test. The correlation of gene expression was evaluated by Spearman’s correlation and statistical significance, and the strength of the correlation was determined using the following criteria: 0.00–0.19 “very weak,” 0.20–0.39 “weak,” 0.40–0.59 “moderate,” 0.60–0.79 “strong,” 0.80–1.0 “very strong.” *P* -value<0.05 was considered statistically significant.

## Results

### Assessment of *TTN* Expression and Biological Functions in Different Tumors and Normal Tissues

**Figure 1:**
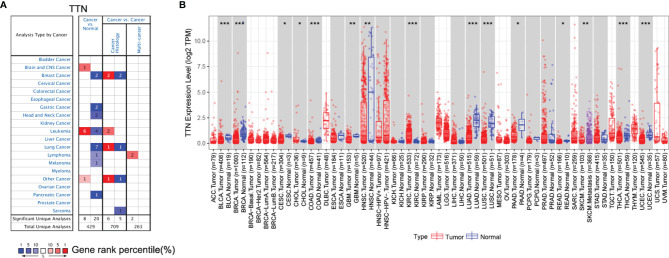
*TTN* gene expression level in different cancer and normal tissues. **(A)** The expression level of *TTN* in different cancer and normal tissues in the Oncomine database. Cell color was determined by the best gene rank percentile for the analyses within the cell; **(B)** The expression of *TTN* between tumor tissues and normal tissues in different cancer types in TIMER database (*P <0.05, **P < 0.01, ***P < 0.001).

Oncomine database was used to determine the expression of *TTN* in various types of cancer and normal tissues. mRNA expression of *TTN* was significantly lower in lung cancer (**Figure 1A**). The panel shows the numbers of datasets with statistically significant mRNA upregulated expression (red) or downregulated expression (blue) of *TTN*. The threshold was designed with the following parameters: fold change of 1.5 and P-value of 0.05.

Furthermore, we evaluated the expression of *TTN* in different types of cancer using the RNA-seq data of multiple malignancies in TCGA. The results manifested that tumor tissues had a significantly decreased *TTN* expression compared with that in normal tissues in LUAD (**Figure 1B**). In addition, it revealed the same tendency both in the microarray and RNA-seq data.

### Prognosis Analysis in LUAD Patients With Different *TTN* Expression Levels

**Figure 2:**
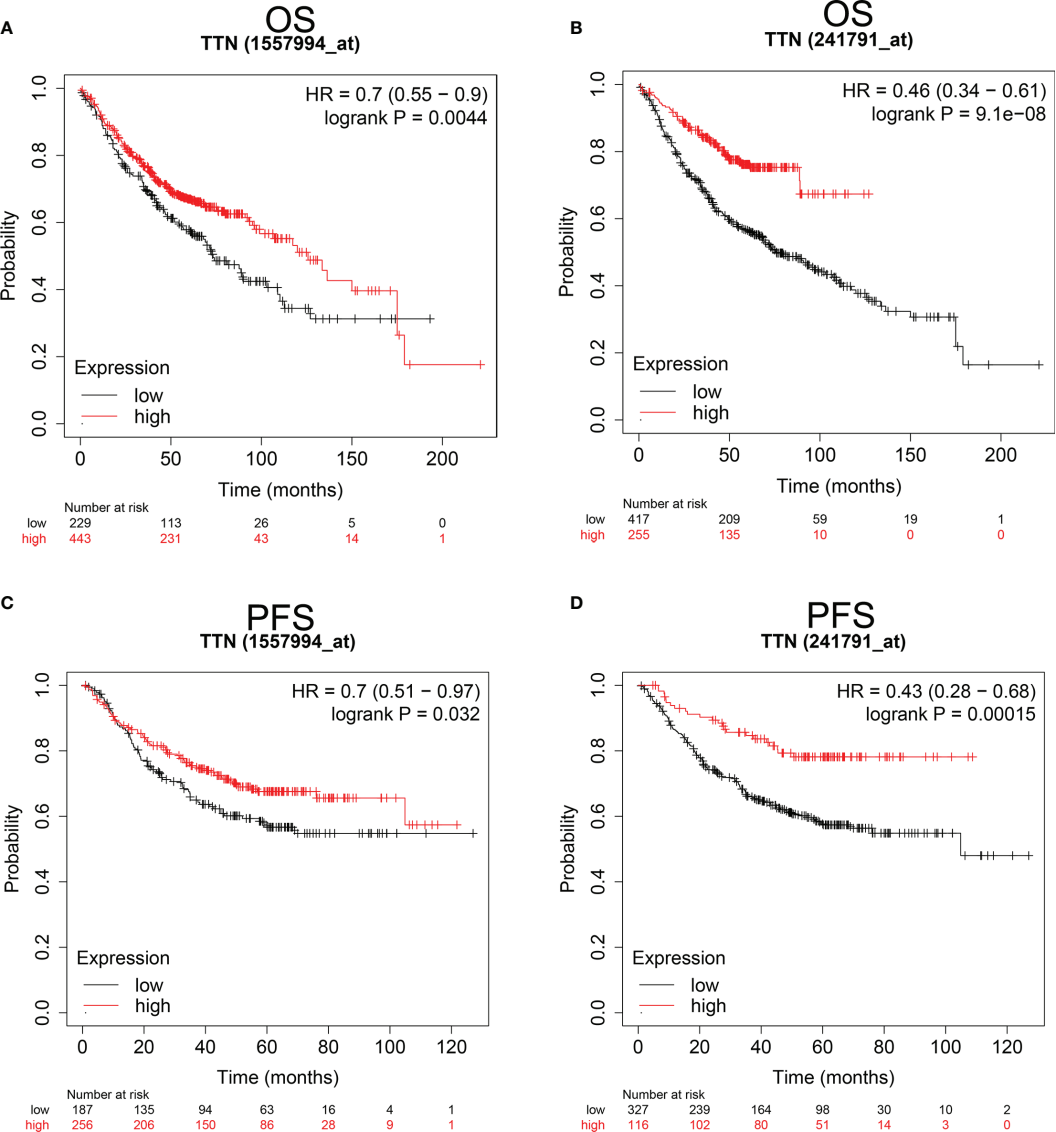
Kaplan-Meier survival curves comparing the higher and lower expression of *TTN* in LUAD Kaplan-Meier plotter database. OS and PFS of LUAD patients in the Kaplan-Meier plotter database **(A–D)**. Red curves represented patients with higher expression of *TTN*. OS, overall survival; RFS, relapse-free survival. PFS, progress-free survival.

Since *TTN* expression was significantly changed in various tumor tissues, especially in LUAD, we investigated the Prognostic value of *TTN* expression in LUAD patients (**Figure 2**). Kaplan-Meier analysis revealed that higher expression of *TTN* predicted better overall survival (OS) and progress-free survival (PFS). (OS HR= 0.7, 95% CI = 0.55 to 0.9, P=0.0044; PFS HR=0.7, 95% CI = 0.51 to 0.97, P=0.032) (OS HR= 0.46, 95% CI = 0.34 to 0.61, P = 9.1e-8; PFS HR = 0.43, 95% CI = 0.28 to 0.68, P = 0.00015) (**Figures 2A–D**). Therefore, lower expression level of *TTN* may serve as a poor prognostic factor in LUAD patients.

### Correlation of *TTN* Expression With Prognosis Under Different Clinicopathological Factors in LUAD Patients

**Figure 3:**
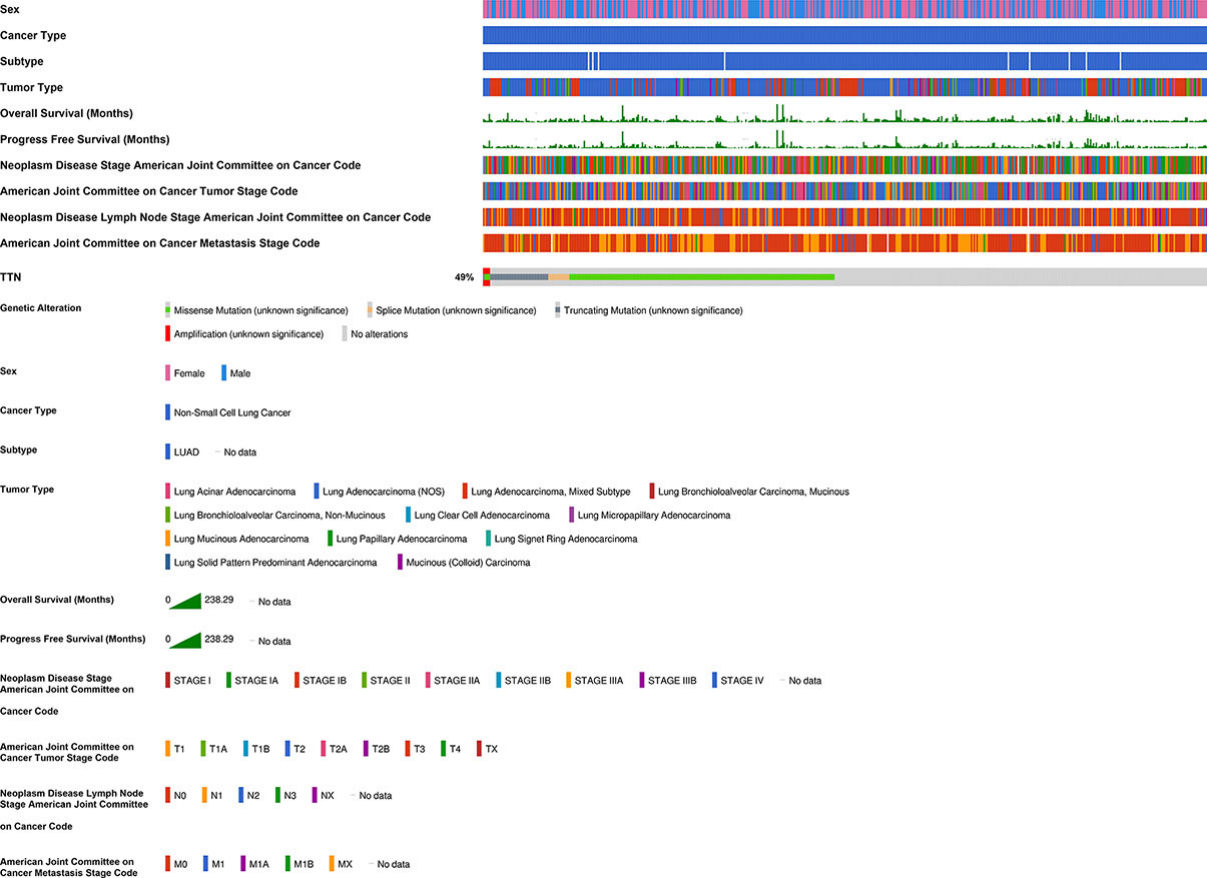
Correlation of *TTN* mRNA expression with different clinicopathological features in LUAD in the cBioPortal database. The mutation frequency of *TTN* was 49% in 511 patients with LUAD. 55 patients were excluded from analysis since they were not profiled for all queried genes in all queried profiles and germline mutations.

We investigated the relationship between *TTN* expression level with several clinicopathological features and prognosis in LUAD patients in the Kaplan-Meier plotter database (**Table 1**). Besides, the correlation between *TTN* expression and various characteristics in LUAD patients was visualized by the cBioPortal online tool (**Figure 3**). *TTN* mutation frequently occurs in LUAD patients with a rate of 49%. *TTN* missense mutation was the most common type of mutation which caused decreased mRNA expression (**Figure S1**). Moreover, high expression of *TTN* in female patients can benefit in PFS (n = 221, HR = 0.51, 95% CI = 0.32 to 0.83, P = 0.0055) and OS (n = 286, HR = 0.59, 95% CI = 0.4 to 0.89, P = 0.01) while male patients with high *TTN* expression level can benefit in OS (n = 328, HR = 0.62, 95% CI = 0.44 to 0.86, P = 0.0041). Thus, these results provided a theoretical basis for the poor prognosis of patients with *TTN* mutations.

**Table 1:** Correlation between *TTN* mRNA expression and clinical prognosis in LUAD with different clinicopathological factors by Kaplan-Meier plotter.

Clinicopathological characteristics		Overall survival (n =672)			Progression-free survival (n =443)		
		N	Hazard ratio	P-value	N	Hazard ratio	P-value
**Gender**	**Male**	328	0.62(0.44-0.86)	0.0041	222	1.44(0.91-2.28)	0.11
	**Female**	286	0.59(0.4-0.89)	0.01	221	0.51(0.32-0.83)	0.0055
**Stage**	**1**	346	0.56(0.37-0.83)	0.0035	274	0.58(0.34-0.97)	0.035
	**2**	118	0.6(0.34-1.05)	0.07	98	0.69(0.34-1.38)	0.29
	**3**	21	0.17(0.04-0.77)	0.0093	8		
**AJCC stage T**	**1**	115	0.42(0.22-0.83)	0.0095	41	0.2(0.02-1.82)	0.11
	**2**	93	1.41(0.77-2.57)	0.27	81	0.5(0.24-1.02)	0.053
**AJCC stage N**	**0**	174	0.52(0.31-0.88)	0.014	92	0.46(0.2-1.06)	0.061
	**1**	37	0.59(0.24-1.48)	0.26	32	3.84(0.85-17.33)	0.061
**AJCC stage M**	**0**	211	0.59(0.38-0.93)	0.02	124	0.63(0.32-1.26)	0.19
**Smoking history**	**Never**	140	0.29(0.13-0.66)	0.0017	140	0.41(0.22-0.78)	0.0045
	**Smoke**	231	0.66(0.4-1.06)	0.086	229	1.35(0.85-2.12)	0.2

### *TTN* Expression Level and Function in Different Single Cell Type Clusters of Lung Tissue

**Figure 4:**
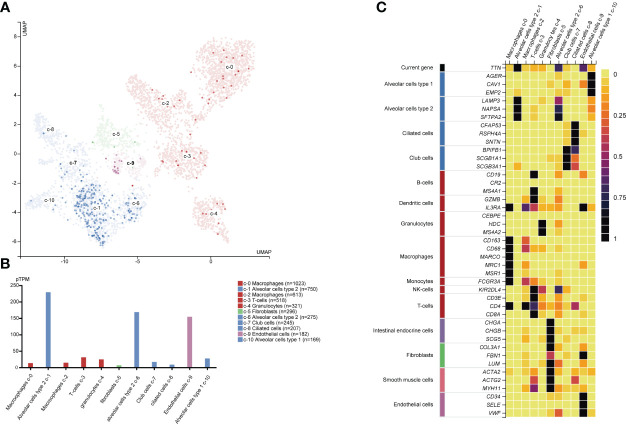
*TTN* mRNA expression and other related cell type markers in different single cell type clusters of lung tissue. Expression of *TTN* in the single-cell type clusters identified in lung tissue was visualized by a UMAP plot **(A)** and a bar chart **(B)** Each dot corresponds to a cell. The heatmap **(C)** of *TTN* and specific marker genes in each cell cluster subtype.

**Figure 5:**
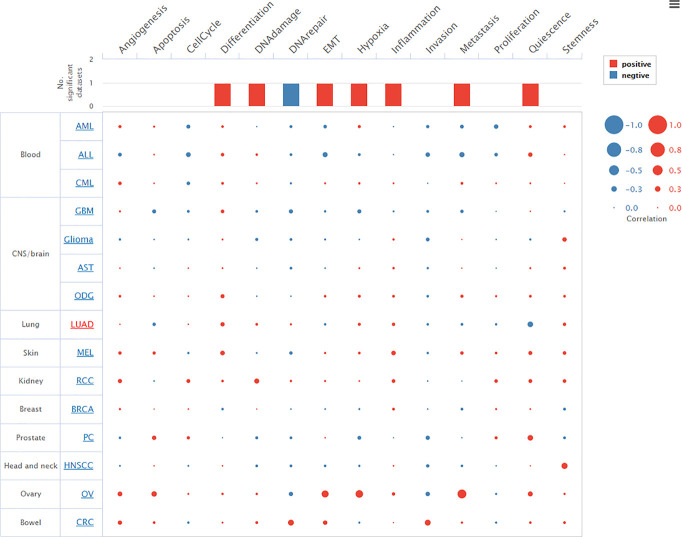
Relevance of *TTN* across 14 functional states in distinct cancers. Average correlations between *TTN* and functional states in different cancers. The bar chart showed the number of datasets in which *TTN* was significantly related to the corresponding state. The red plots indicated that *TTN* was positively correlated with the functional state while the blue plots indicated that *TTN* was negatively correlated with the functional state.

We investigated the transcriptomic expression level in diverse cell clusters of lung tissue (**Figure 4**). The results showed each value in different cluster, and we found that *TTN* had a higher expression level in alveolar cells type 2 c-1(n=750, 228.9pTPM), c-6 (n=275, 168.4pTPM) and endothelial cells c-9 (n=182, 154.2pTPM). Consequently, *TTN* function in tumorigenesis was investigated in nine organs or tissues at a single-cell level, including LUAD and other tumors (**Figure 5**). The results showed that *TTN* had a negative correlation with DNA repair function, and it also significantly related to differentiation in LUAD (ρ=0.37, p<0.01).

### *TTN* Expression Correlated With Immune Cell Infiltration in LUAD

**Figure 6:**
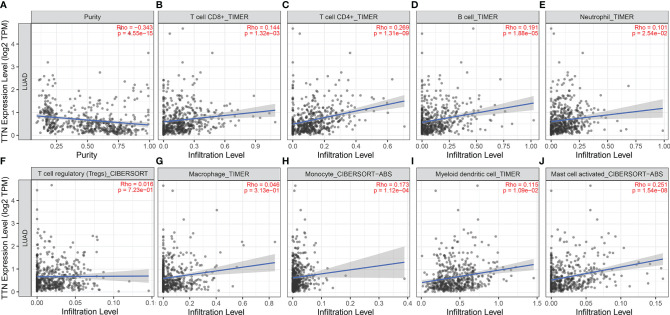
Correlations between TTN expression level and tumor-infiltrating immune cells in LUAD. *TTN* expression displayed significant correlations with tumor purity **(A)** and infiltration of CD8+T cells **(B)**, CD4+T cells **(C)**, B cells **(D)**, neutrophils **(E)**, monocytes **(H)**, DCs **(I)**, and mast cells **(J)** activated in LUAD. *TTN* expression showed a very weak correlation with Treg **(F)** and macrophages **(G)** in LUAD

**Figure 7:**
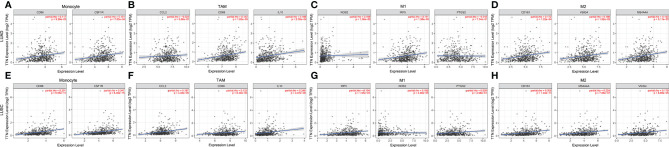
*TTN* expression correlated with macrophage polarization in NSCLC. Scatterplots of correlations between *TTN* expression and gene markers of monocytes **(A)**, TAMs **(B)** and M1 **(C)** and M2 macrophages **(D)** in LUAD(n=515) **(A–D)**. Scatterplots of correlations between *TTN* expression and gene markers of monocytes **(E)**, TAMs **(F)** and M1 **(G)** and M2 macrophages **(H)** in LUSC(n=501) **(E–H)**.

We investigated the association between *TTN* expression and infiltration level of immune cells in the tumor microenvironment (**Figure 6**). *TTN* expression level was positively correlated with the infiltration of CD8+ T cells (Rho = 0.144, P = 1.32e-03), CD4+ T cells (Rho = 0.269, P = 1.31e-09), B cells (Rho = 0.191, P = 1.88e-05), neutrophils (Rho = 0.101, P = 2.54e-02), T cell regulatory (Rho = 0.016, P = 7.23e-01), macrophages (Rho = 0.046, P = 3.13e-01), monocytes (Rho = 0.173, P = 1.12e-04), myeloid dendritic cells (Rho = 0.115, P = 1.09e-02) and mast cell activated (Rho = 0.251, P = 1.54e-08). The results showed that *TTN* and different tumor-infiltrating immune cell subsets were weakly to moderately correlated. Biomarker sets of immune cells were significantly associated with *TTN* in LUAD (**Table S1**). Markers of monocyte and macrophage were significantly correlated with the expression level of *TTN* in TIMER and GEPIA databases. (**Figure 7** and **Table 2**). Moreover, *TTN* had the highest correlation with T cells and its markers implicated the potential of *TTN* to recruit and activate T cells. Above all, these results indicated that *TTN* played a potentially important role in modulating the immune microenvironment of LUAD.

**Table 2:** Correlation analysis between *TTN* and related genes and markers of monocyte and macrophages in GEPIA.

Description	Gene markers	LUAD				LUSC			
		Tumor		Normal		Tumor		Normal	
		R	P	R	P	R	P	R	P
**Monocyte**	**CD86**	0.22	***	-0.044	0.74	0.11	*	0.089	0.54
	**CSF1R**	0.29	***	0.1	0.43	0.19	***	0.013	0.93
**TAM**	**CCL2**	0.12	**	-0.038	0.78	0.083	0.068	-0.081	0.58
	**CD68**	0.21	***	-0.13	0.33	0.02	0.66	0.069	0.63
	**IL10**	0.25	***	-0.097	0.47	0.073	0.11	-0.24	0.091
**M1 Macrophage**	**IRF5**	0.25	***	-0.011	0.93	0.11	*	0.1	0.49
	**NOS2**	0.15	**	0.12	0.35	0.15	***	0.1	0.47
	**PTGS2**	0.059	0.19	0.23	0.075	0.11	*	0.038	0.79
**M2 macrophage**	**CD163**	0.23	***	-0.074	0.58	0.14	**	-0.094	0.52
	**MS4A4A**	0.21	***	-0.16	0.22	0.074	0.1	-0.067	0.64
	**VSIG4**	0.16	***	-0.11	0.4	0.032	0.48	0.26	0.071

LUAD, lung adenocarcinoma; LUSC, lung squamous cell carcinoma; TAM, Tumor-associated macrophages; Tumor, correlation analysis in tumor tissue of TCGA; Normal, correlation analysis in normal tissue of TCGA. (*P < 0.05; **P < 0.01; ***P < 0.001).

### Verification of *TTN* Prognostic Value and Expression

**Figure 8:**
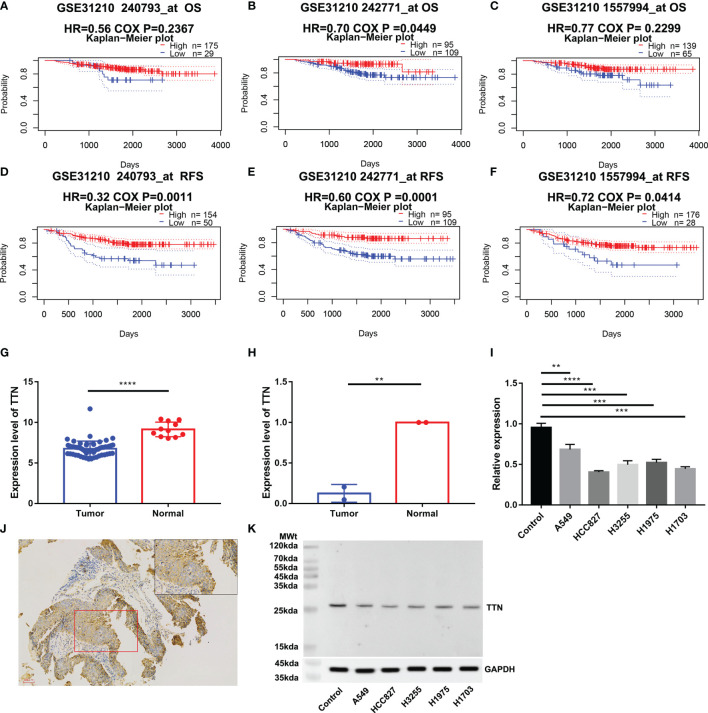
Validation of *TTN* expression and prognostic value. OS and RFS of LUAD patients in the cohort (GSE312100, n=204) **(A–F)**; Expression of *TTN* in cohort (GSE116959, n= 68) **(G)**; *TTN* expression level in 2 LUAD tissue samples and 2 paired normal tissue samples **(H)**; *TTN* expression level in 4 LUAD cell samples,1 LUSC cell sample and 1 normal alveolar epithelial cell sample **(I)**; Immunohistochemical analysis of the expression of titin in LUAD **(J)**. Western blotting analysis of expression of titin in 5 different lung cancer cell lines and human pulmonary alveolar epithelial cells **(K)**. (*P < 0.05, **P < 0.01, ***P < 0.001, ****P < 0.0001)

We investigated the Prognostic value of *TTN* Expression in LUAD in the PrognoScan database. The cohort (GSE31210), including 204 samples, showed that a lower *TTN* expression level was significantly associated with poor prognosis (**Figures 8B, D–F**). However, the cohort (240793_at and 1557994_at) showed no significant association between *TTN* expression and OS in LUAD (**Figures 8A, C**). In an independent (GSE116959), we found that *TTN* had a lower expression level, comparing to normal tissues (**Figure 8G**). In order to validate our findings, we also extracted RNA from 4 paired samples from 2 LUAD patients, 6 samples from five lung cancer cell lines and one normal cell line, and a similar result was also observed (**Figures 8H, I**). Immunohistochemical analysis confirmed that *TTN* expression was lower in LUAD than in normal lung tissue (**Figure 8J**). Western blotting analysis demonstrated that five lung cancer cell samples had significantly decreased expression of titin, compared with normal alveolar epithelial cell sample (**Figure 8K**).

## Discussion

On the basis of our findings and rigorous validation, we found *TTN* was strongly correlated with prognosis and tumor-infiltrating lymphocytes. Both Oncomine and TIMER databases showed consistent differences between tumor and normal tissues, which means *TTN* was a differentially expressed gene and deserved further study. Subsequently, we carried out prognosis analysis in LUAD patients with different *TTN* expression levels. Results in our findings indicated that lower expression levels in LUAD boded poor outcomes. In addition, we *de novo* verified our findings through two independent cohorts, qPCR, and immunohistochemistry methods. Thus, it could be a prognostic biomarker for LUAD patients.

Based on previous knowledge, we have learned that Alveolar Type 2 (AT2) cells are precursors of the alveolar epithelium, which can transform into AT1 cells, maintain self-renewal during normal homeostasis and recovery after injury (35–37). Some investigators found that AT2 cells may progress to adenomas and adenocarcinomas (38–40). The pathogenesis of early adenocarcinoma progression indicated that AT2 cells were the most likely cancer cells of origin (41). Recent advances in Single-cell RNA sequencing (scRNA-seq) revealed AT2-like cells were associated with malignant cell populations, and AT2 cells were regarded as the cancer-initiating cells (42, 43). According to our findings, *TTN* was highly expressed in AT2 cells at single-cell resolution (**Figure 4**). Meanwhile, a negative correlation between *TTN* with DNA repair was exclusively observed in the CancerSEA database (**Figure 5**). As we all know, DNA repair dysfunction could lead to tumorigenesis, which means *TTN* may be a potential determinant in the development of cancer.

Another important result in our study was the correlation between *TTN* expression and tumor-infiltrating lymphocytes. *TTN* expression level was positively correlated with the infiltration levels of CD8+ T cells, CD4+ T cells, and other immune cells (**Figure 6**). The tumor-infiltrating lymphocyte grade is a key factor in tumor staging and is one of the cogent predictors of cancer recurrence and survival (44, 45). This result may help explain the relationship between the low expression level of *TTN* and poor survival rate in patients with LUAD. Our study also showed that *TTN* significantly and specifically correlated with PD-1, CTLA-4, LAG-3, TIM-3. We can infer that *TTN* may have an influence on the clinical outcome of LUAD patients by means of interacting with immune infiltrating cells. Effective biomarkers which could improve response rates of immunotherapy are the basis of drug development and clinical precision medicine. Our findings also provided a tendency in scientific research and clinical use of immunotherapy in the future.

*TTN* was frequently detected with a high mutation rate in solid tumors, and was associated with responsiveness to checkpoint blockades in solid tumors (46). Furthermore, the detection of *TTN* mutations in peripheral blood was pertinent to satisfactory objective response and survival rate of ICBs (47). Recent studies also showed that *TTN* mutation has great potential as a predictive marker of ICBs for LUAD patients (48).

This study is the first research to explore the transcription level, function states of *TTN*, and its relationship with the prognosis and immune infiltration in patients with LUAD through bioinformatics analysis and validation *in vitro*, which may provide a potential biomarker for immunotherapy. Besides, our findings shed valuable insights on the roles of *TTN* in AT2 cell tumorigenesis. However, our study has several limitations. The precise role of *TTN* in tumor immune microenvironment is not clear, and the animal model is still needed to elucidate the underlying mechanism that how *TTN* promotes evolution and dissemination of tumors through mediating tumor-infiltrating immune cells.

In conclusion, our research indicates that the transcription level of *TTN* is obviously downregulated in LUAD and may play a significant role in the occurrence and development of LUAD. Besides, *TTN* has the potential as an immune-related therapeutic target.

## Data Availability Statement

The original contributions presented in the study are included in the article/**Supplementary Material**. Further inquiries can be directed to the corresponding author.

## Ethics Statement

The studies involving human participants were reviewed and approved by the ethics committee of Shanghai Pulmonary Hospital, Tongji University. The patients/participants provided their written informed consent to participate in this study.

## Author Contributions

JC: Data analysis, Interpretation, Manuscript writing; YW: Collection and data assembly, Manuscript writing; HS: Provision of study materials or patients, Manuscript writing; XY: Manuscript writing. RH: Manuscript revision. CC: Conceptualization, Manuscript Revision; CS: Conceptualization, Administrative support, Manuscript Revision. All authors have reviewed the final version of the manuscript and approve it for publication.

## Funding

This study was supported by the National Natural Science Foundation of China (grant number: 82072568) and the Cancer Research Funding of CSCO-Hausen (grant number: kh0151120201494, kh0151120200974).

## Conflict of Interest

The authors declare that the research was conducted in the absence of any commercial or financial relationships that could be construed as a potential conflict of interest.

## Publisher’s Note

All claims expressed in this article are solely those of the authors and do not necessarily represent those of their affiliated organizations, or those of the publisher, the editors and the reviewers. Any product that may be evaluated in this article, or claim that may be made by its manufacturer, is not guaranteed or endorsed by the publisher.

## Supplementary Material

Supplementary Figure 1 mRNA expression of different *TTN* mutation types. Missense mutation has decreased slightly with no statistical significance. P = 0.1642.
