# Supplementary figures and images for: Deciphering Prognostic Value of TTN and Its Correlation With Immune Infiltration in Lung Adenocarcinoma
Source: Front Oncol. 2022 Jul 8;12:877878. doi: 10.3389/fonc.2022.877878 (PMC9304871; doi:10.3389/fonc.2022.877878)

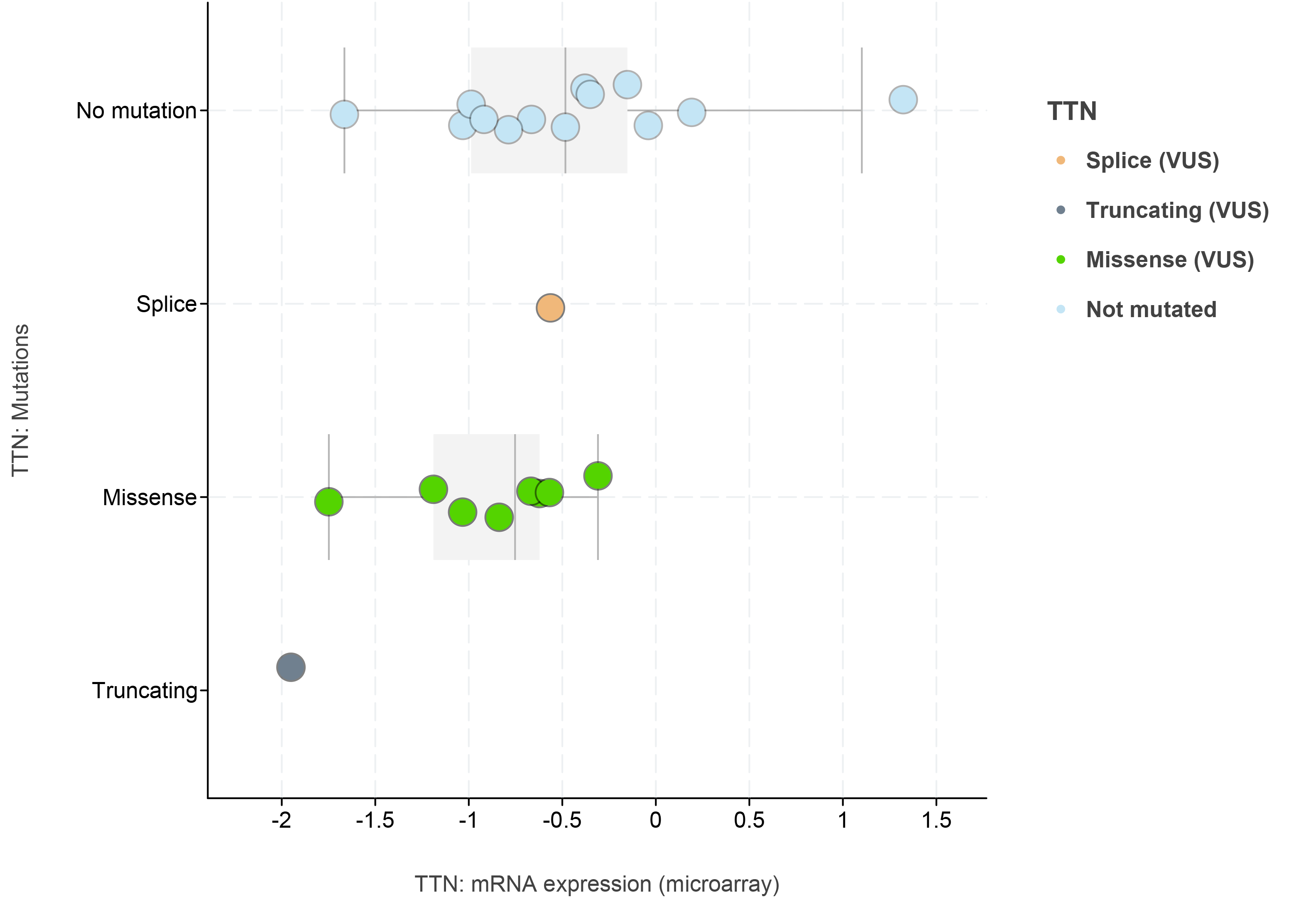

Supplement: Supplementary Figure 1 — mRNA expression of different TTN mutation types. Missense mutation has decreased slightly with no statistical significance. P = 0.1642. [file Image_1.tif]

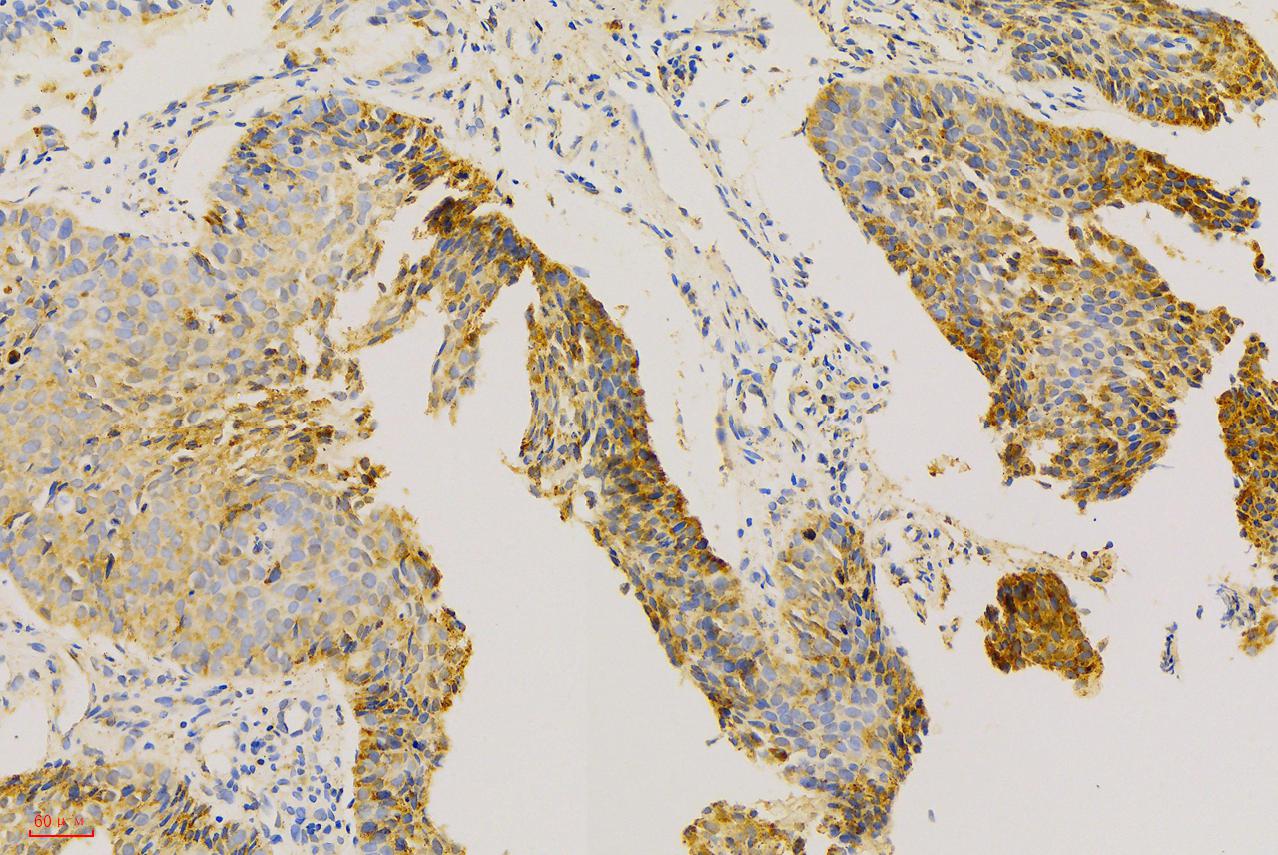

Supplement: Supplementary file 2 [file Image_2.jpeg]

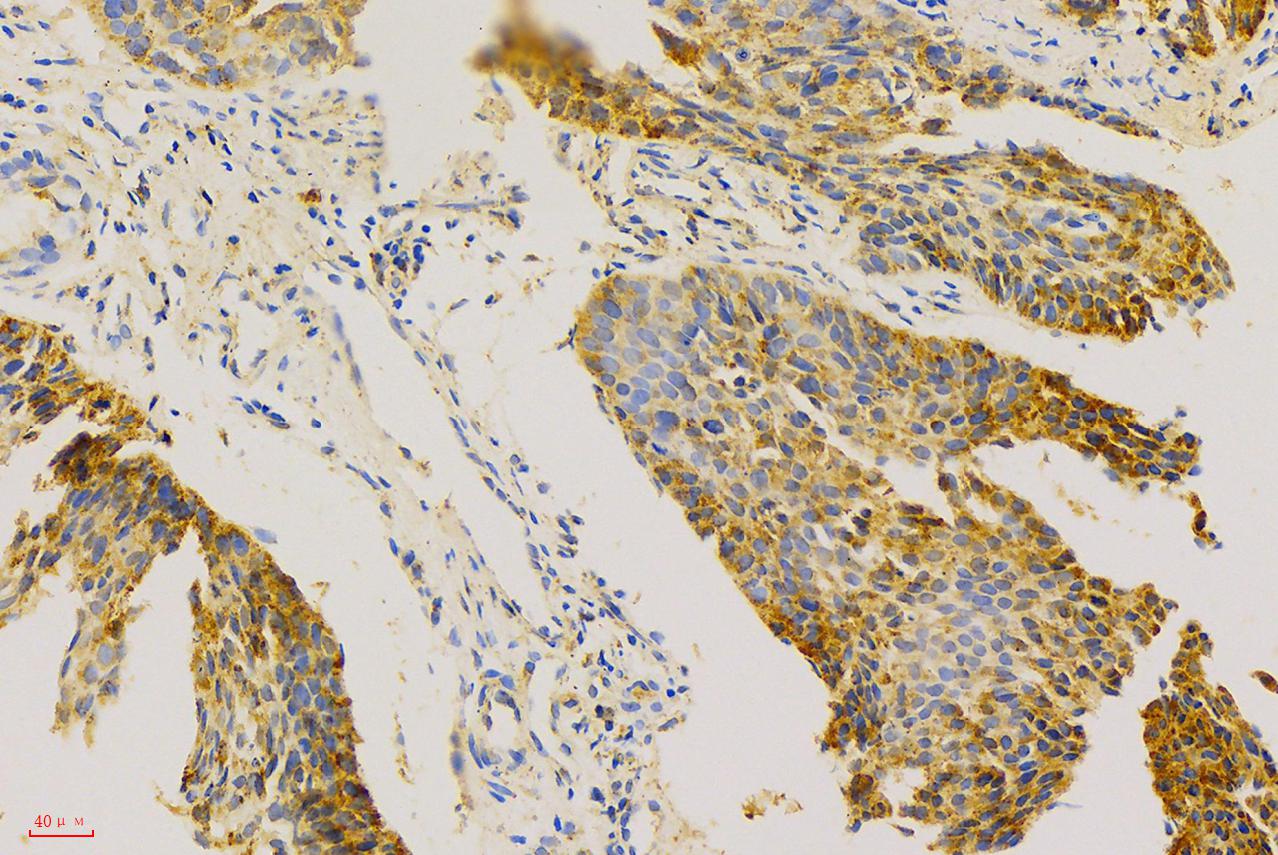

Supplement: Supplementary file 3 [file Image_3.jpeg]

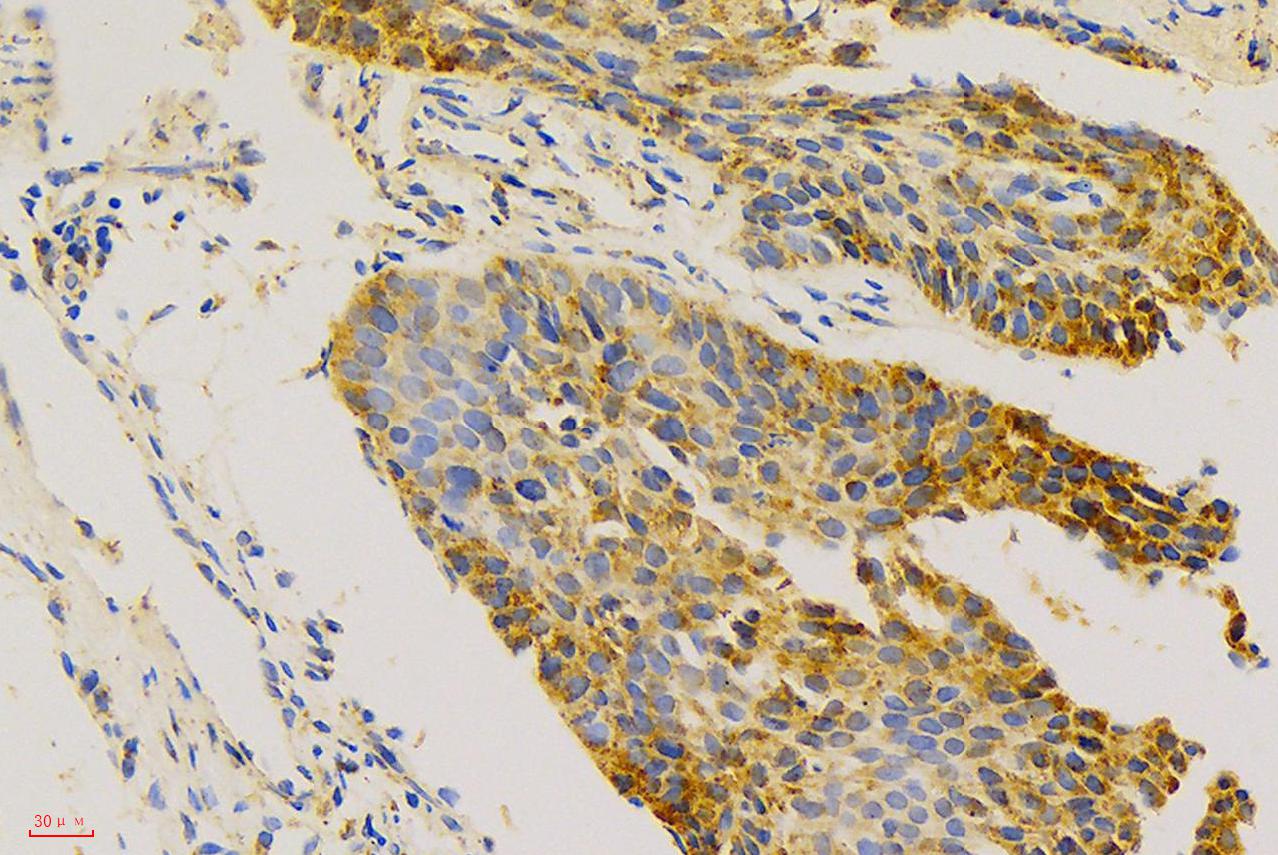

Supplement: Supplementary file 4 [file Image_4.jpeg]

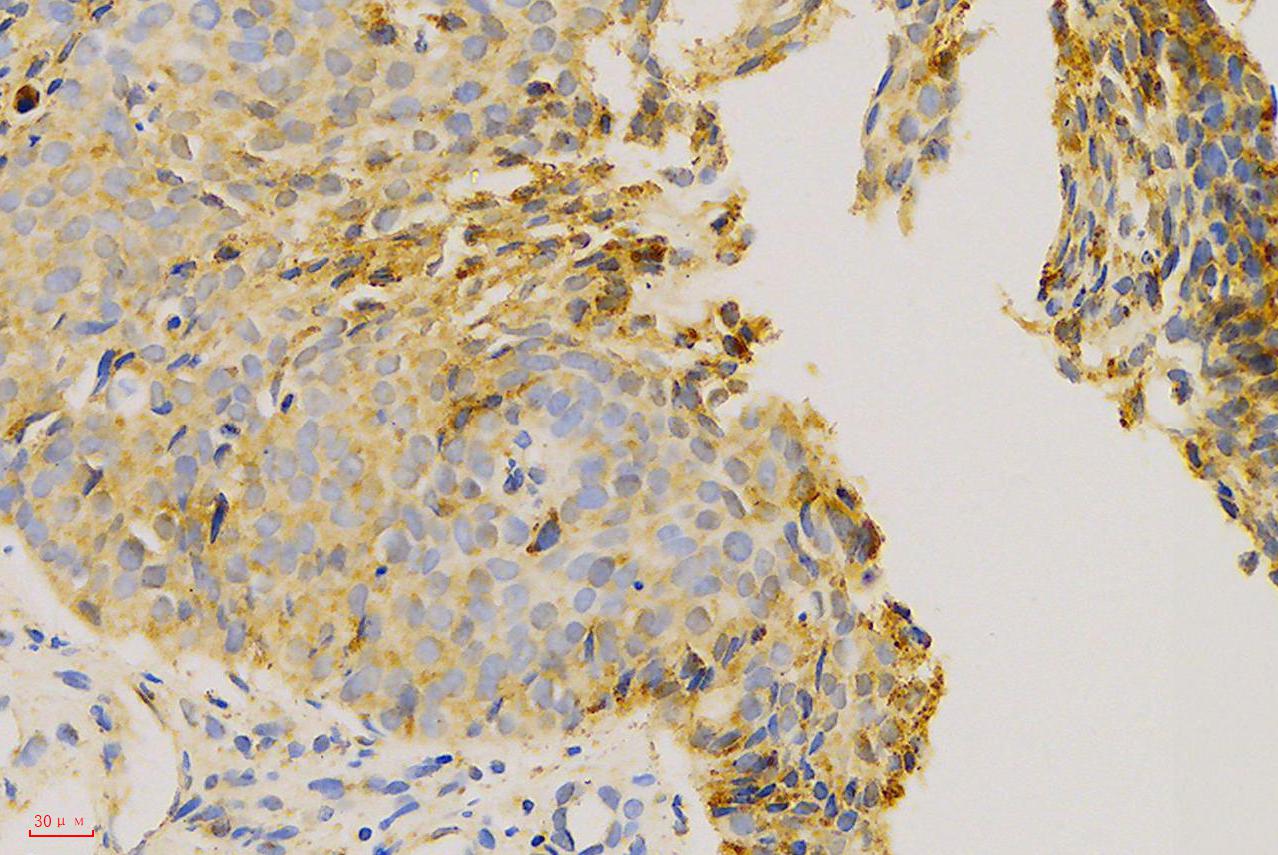

Supplement: Supplementary file 5 [file Image_5.jpeg]

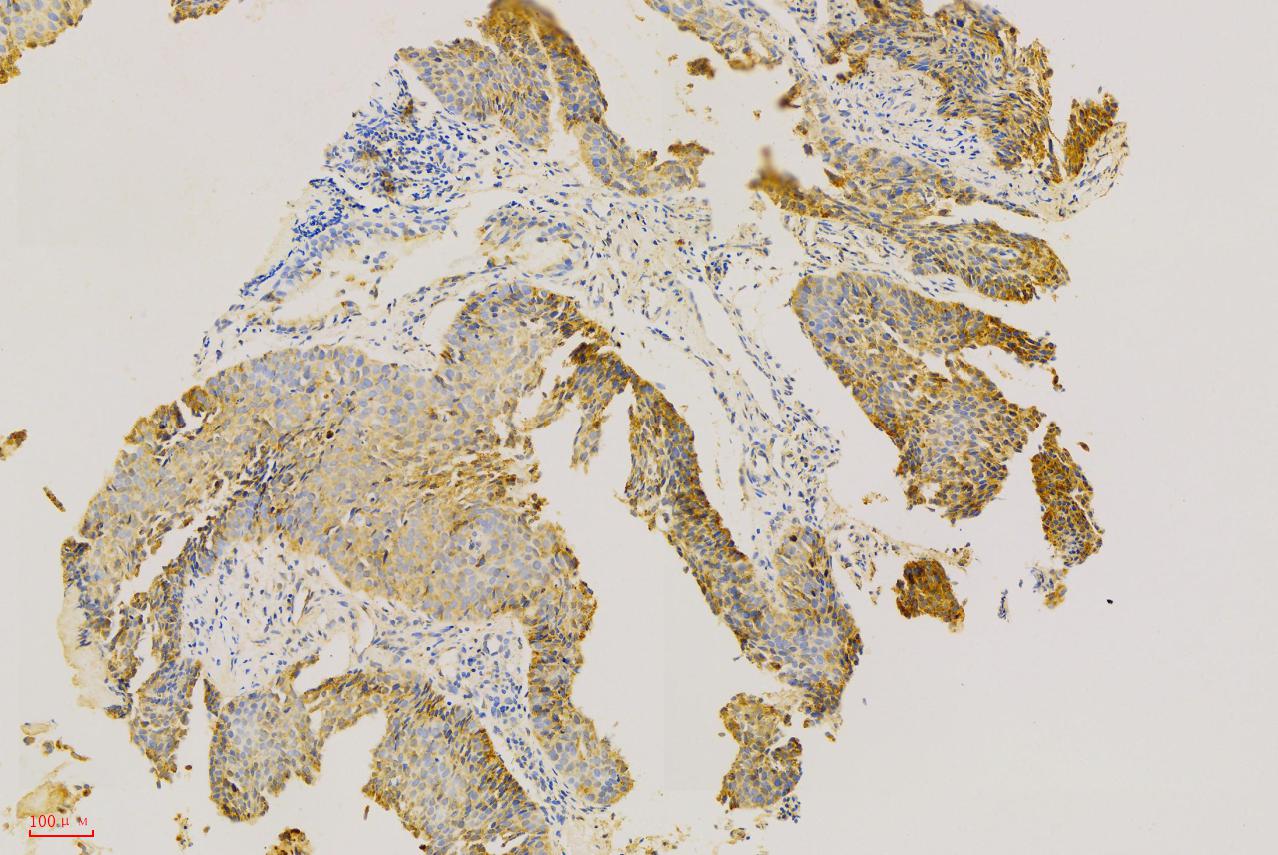

Supplement: Supplementary file 6 [file Image_6.jpeg]

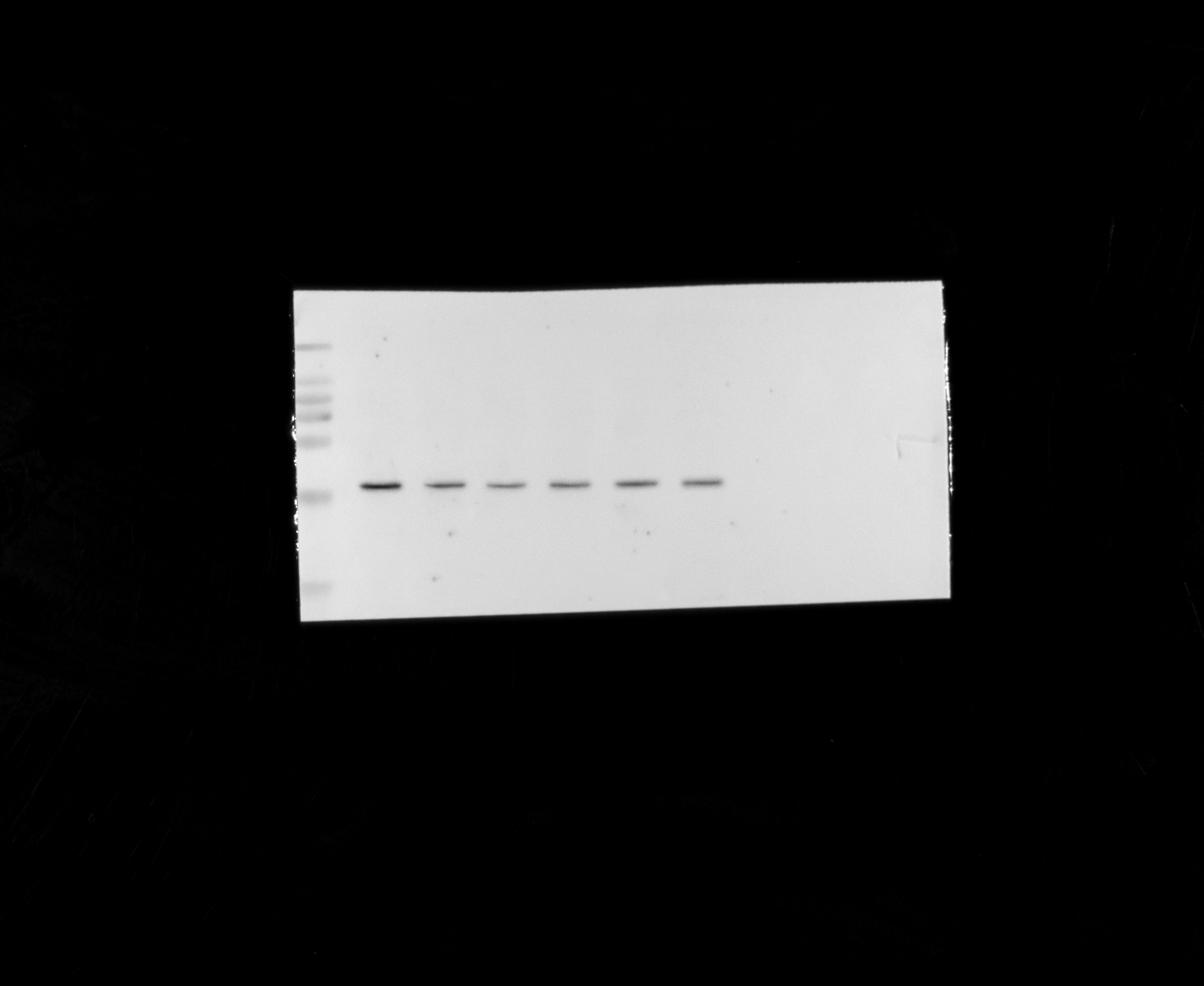

Supplement: Supplementary file 7 [file Image_7.tif]

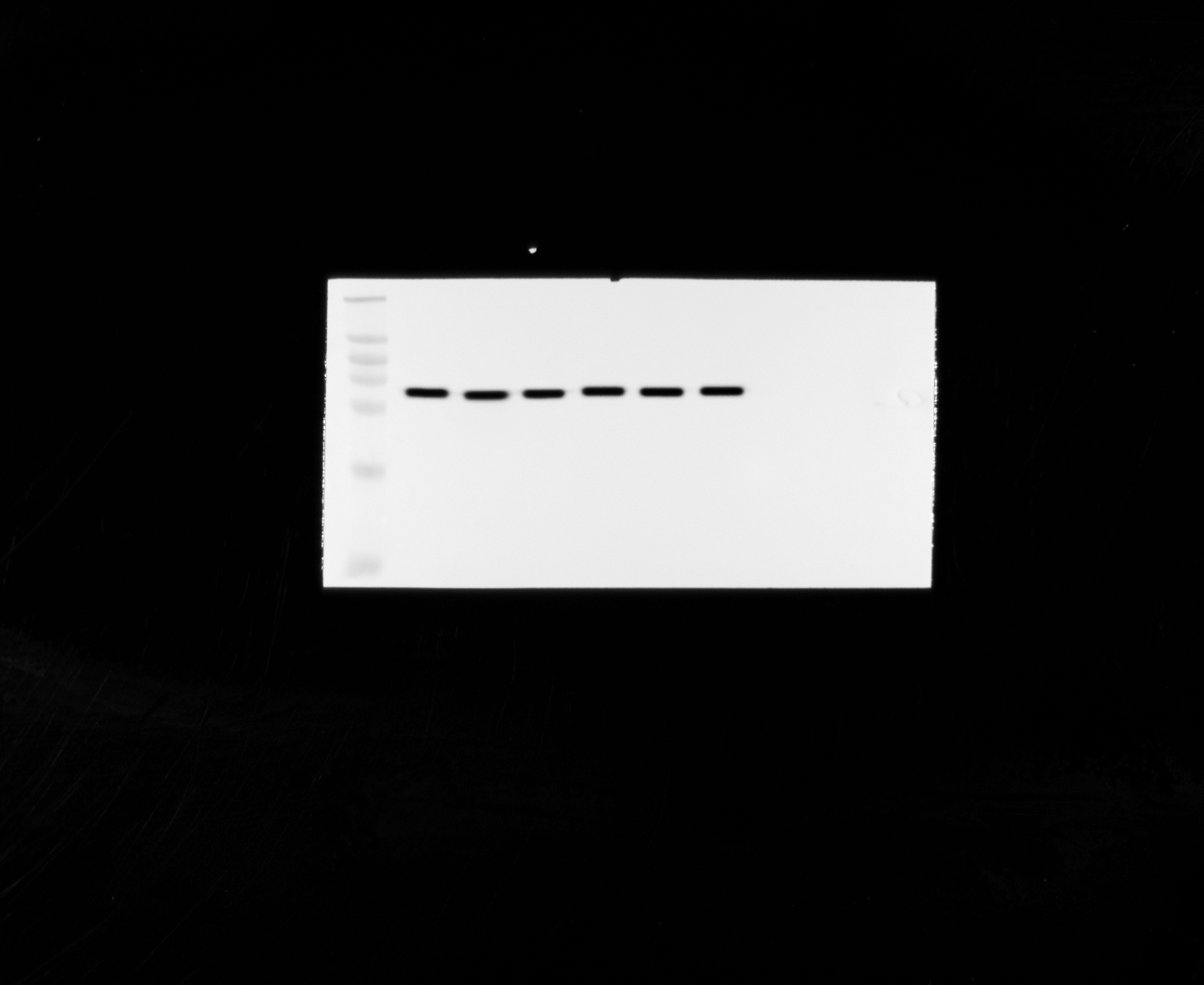

Supplement: Supplementary file 8 [file Image_8.tif]
