# Supplementary material for: Deciphering Prognostic Value of TTN and Its Correlation With Immune Infiltration in Lung Adenocarcinoma
Source: Front Oncol. 2022 Jul 8;12:877878. doi: 10.3389/fonc.2022.877878 (PMC9304871; doi:10.3389/fonc.2022.877878)
Supplement: Supplementary file 9 [file Table_1.docx]

**Table S1** Correlation analysis between TTN and related gene markers of immune cells in TIMER.

| **Description** | **Gene markers** | **LUAD** | | | | **LUSC** | | | |
| --- | --- | --- | --- | --- | --- | --- | --- | --- | --- |
|  |  | **None** | | **Purity** | | **None** | | **Purity** | |
|  |  | **Cor** | **P** | **Cor** | **P** | **Cor** | **P** | **Cor** | **P** |
| **CD8+ T cell** | **CD8A** | 0.370 | *** | 0.267 | *** | 0.390 | *** | 0.342 | *** |
|  | **CD8B** | 0.305 | *** | 0.215 | *** | 0.323 | *** | 0.291 | *** |
| **T cell (general)** | **CD2** | 0.440 | *** | 0.329 | *** | 0.435 | *** | 0.384 | *** |
|  | **CD3D** | 0.376 | *** | 0.247 | *** | 0.399 | *** | 0.342 | *** |
|  | **CD3E** | 0.476 | *** | 0.375 | *** | 0.451 | *** | 0.402 | *** |
| **B cell** | **CD19** | 0.400 | *** | 0.294 | *** | 0.422 | *** | 0.367 | *** |
|  | **CD79A** | 0.281 | *** | 0.162 | *** | 0.333 | *** | 0.255 | *** |
| **Monocyte** | **CD86** | 0.251 | *** | 0.117 | * | 0.350 | *** | 0.291 | *** |
|  | **CSF1R** | 0.264 | *** | 0.150 | ** | 0.400 | *** | 0.347 | *** |
| **TAM** | **CCL2** | 0.103 | * | -0.246 | 0.634 | 0.237 | *** | 0.187 | *** |
|  | **CD68** | 0.248 | *** | 0.142 | ** | 0.193 | *** | 0.125 | *** |
|  | **IL10** | 0.290 | *** | 0.166 | *** | 0.295 | *** | 0.248 | *** |
| **M1 Macrophage** | **IRF5** | 0.284 | *** | 0.191 | *** | 0.199 | *** | 0.194 | *** |
|  | **NOS2** | 0.174 | *** | 0.109 | * | 0.168 | *** | 0.185 | *** |
|  | **PTGS2** | -0.022 | 0.768 | -0.015 | 0.823 | 0.087 | 0.1 | 0.039 | 0.519 |
| **M2 macrophage** | **CD163** | 0.279 | *** | 0.172 | *** | 0.363 | *** | 0.305 | *** |
|  | **MS4A4A** | 0.258 | *** | 0.133 | ** | 0.292 | *** | 0.224 | *** |
|  | **VSIG4** | 0.210 | *** | 0.106 | * | 0.246 | *** | 0.179 | *** |
| **Neutrophils** | **CCR7** | 0.491 | *** | 0.398 | *** | 0.515 | *** | 0.482 | *** |
|  | **CEACAM8** | 0.165 | *** | 0.162 | ** | 0.161 | *** | 0.155 | ** |
|  | **ITGAM** | 0.305 | *** | 0.200 | *** | 0.453 | *** | 0.412 | *** |
|  | **KIR2DL1** | 0.240 | *** | 0.209 | *** | 0.163 | *** | 0.127 | * |
|  | **KIR2DL3** | 0.204 | *** | 0.133 | ** | 0.221 | *** | 0.189 | ** |
|  | **KIR2DL4** | 0.118 | * | 0.050 | 0.371 | 0.188 | *** | 0.150 | *** |
|  | **KIR2DS4** | 0.248 | *** | 0.201 | *** | 0.204 | *** | 0.184 | *** |
|  | **KIR3DL1** | 0.267 | *** | 0.226 | *** | 0.254 | *** | 0.216 | *** |
|  | **KIR3DL2** | 0.201 | *** | 0.133 | ** | 0.223 | *** | 0.182 | *** |
|  | **KIR3DL3** | 0.094 | 0.056 | 0.082 | 0.125 | 0.030 | 0.586 | 0.021 | 0.741 |
| **Dendritic cell** | **CD1C** | 0.288 | *** | 0.209 | *** | 0.336 | *** | 0.277 | *** |
|  | **HLA-DPA1** | 0.334 | *** | 0.249 | *** | 0.440 | *** | 0.389 | *** |
|  | **HLA-DPB1** | 0.400 | *** | 0.313 | *** | 0.463 | *** | 0.414 | *** |
|  | **HLA-DQB1** | 0.301 | *** | 0.198 | *** | 0.328 | *** | 0.267 | *** |
|  | **HLA-DRA** | 0.301 | *** | 0.194 | *** | 0.389 | *** | 0.330 | *** |
|  | **ITGAX** | 0.466 | *** | 0.379 | *** | 0.497 | *** | 0.465 | *** |
|  | **NRP1** | 0.059 | 0.207 | 0.023 | 0.662 | 0.203 | *** | 0.141 | ** |
| **Th1** | **IFNG** | 0.250 | *** | 0.150 | ** | 0.304 | *** | 0.265 | *** |
|  | **STAT1** | 0.172 | *** | 0.076 | 0.114 | 0.247 | *** | 0.211 | *** |
|  | **STAT4** | 0.394 | *** | 0.289 | *** | 0.495 | *** | 0.453 | *** |
|  | **TBX21** | 0.514 | *** | 0.424 | *** | 0.520 | *** | 0.483 | *** |
|  | **TNF** | 0.245 | *** | 0.117 | * | 0.259 | *** | 0.211 | *** |
| **Th2** | **GATA3** | 0.317 | *** | 0.217 | *** | 0.337 | *** | 0.290 | *** |
|  | **IL13** | 0.217 | *** | 0.151 | ** | 0.344 | *** | 0.318 | *** |
|  | **STAT5A** | 0.427 | *** | 0.337 | *** | 0.500 | *** | 0.462 | *** |
|  | **STAT6** | 0.280 | *** | 0.304 | *** | 0.331 | *** | 0.347 | *** |
| **Tfh** | **BCL6** | 0.181 | *** | 0.205 | *** | 0.269 | *** | 0.312 | *** |
|  | **IL21** | 0.161 | *** | 0.110 | * | 0.291 | *** | 0.257 | *** |
| **Th17** | **Il17A** | 0.198 | *** | 0.139 | ** | 0.098 | 0.067 | 0.057 | 0.352 |
|  | **STAT3** | 0.061 | 0.263 | 0.080 | 0.160 | 0.276 | *** | 0.257 | **** |
| **Treg** | **CCR8** | 0.288 | *** | 0.178 | *** | 0.364 | *** | 0.314 | *** |
|  | **FOXP3** | 0.290 | *** | 0.160 | *** | 0.400 | *** | 0.347 | *** |
|  | **STAT5B** | 0.343 | *** | 0.333 | *** | 0.353 | *** | 0.377 | *** |
|  | **TGFB1** | 0.244 | *** | 0.155 | ** | 0.033 | *** | -0.019 | *** |
| **T cell exhaustion** | **CTLA4** | 0.432 | *** | 0.322 | *** | 0.470 | *** | 0.430 | *** |
|  | **GZMB** | 0.198 | *** | 0.082 | 0.094 | 0.314 | *** | 0.260 | *** |
|  | **TIM-3** | 0.264 | *** | 0.129 | ** | 0.346 | *** | 0.281 | *** |
|  | **LAG3** | 0.299 | *** | 0.201 | *** | 0.372 | *** | 0.331 | *** |
|  | **PD-1** | 0.357 | *** | 0.252 | *** | 0.480 | *** | 0.437 | *** |

TAM, tumor-associated macrophage; Th, T helper cell; Tfh, Follicular helper T cell; Treg, regulatory T cell; Cor, R value of Spearman’s correlation; None, correlation without adjustment. Purity, correlation adjusted by purity. P<0.05 was considered to be statistically significant. (*p < 0.05, **p < 0.01, ***p < 0.001)
